# Impaired F_1_F_o_-ATP-Synthase Dimerization Leads to the Induction of Cyclophilin D-Mediated Autophagy-Dependent Cell Death and Accelerated Aging

**DOI:** 10.3390/cells10040757

**Published:** 2021-03-30

**Authors:** Verena Warnsmann, Lisa-Marie Marschall, Heinz D. Osiewacz

**Affiliations:** Faculty of Biosciences, Institute of Molecular Biosciences, Goethe University, 60438 Frankfurt, Germany; warnsmann@bio.uni-frankfurt.de (V.W.); L.Marschall@bio.uni-frankfurt.de (L.-M.M.)

**Keywords:** *Podospora anserina*, F_1_F_o_-ATP-synthase, mitophagy, mPTP, ADCD

## Abstract

Mitochondrial F_1_F_o_-ATP-synthase dimers play a critical role in shaping and maintenance of mitochondrial ultrastructure. Previous studies have revealed that ablation of the F_1_F_o_-ATP-synthase assembly factor PaATPE of the ascomycete *Podospora anserina* strongly affects cristae formation, increases hydrogen peroxide levels, impairs mitochondrial function and leads to premature cell death. In the present study, we investigated the underlying mechanistic basis. Compared to the wild type, we observed a slight increase in non-selective and a pronounced increase in mitophagy, the selective vacuolar degradation of mitochondria. This effect depends on the availability of functional cyclophilin D (PaCYPD), the regulator of the mitochondrial permeability transition pore (mPTP). Simultaneous deletion of *PaAtpe* and *PaAtg1*, encoding a key component of the autophagy machinery or of *PaCypD*, led to a reduction of mitophagy and a partial restoration of the wild-type specific lifespan. The same effect was observed in the *PaAtpe* deletion strain after inhibition of PaCYPD by its specific inhibitor, cyclosporin A. Overall, our data identify autophagy-dependent cell death (ADCD) as part of the cellular response to impaired F_1_F_o_-ATP-synthase dimerization, and emphasize the crucial role of functional mitochondria in aging.

## 1. Introduction

Biological aging is a process that is characterized by a time-dependent decrease in physiological functions and an increase in morbidity and mortality of biological systems. It is controlled by environmental, stochastic and genetic traits. Over decades of research, mitochondria have been demonstrated to play a paramount role in aging [1,2,3,4,5,6,7,8]. These organelles are involved in different important cellular processes including energy transduction, iron–sulfur cluster biosynthesis, and programmed cell death (PCD). A network of pathways involved in maintenance of a “healthy” population of mitochondria is effective and counteracts the time-dependent accumulation of functionally impaired mitochondria [9].

*Podospora anserina* is a filamentous fungus with a strain-specific limited lifespan. After germination of an ascospore, the product of sexual propagation, a colony develops consisting of branched filamentous cells (hyphae), which grow at their tips. After a short period of linear growth, growth slows down until it completely stops and the colony dies at the periphery. *P. anserina* is used as a model system to unravel the mechanisms of organismic aging, as reviewed in [10,11,12,13]. Research from the late 1970s and the early 1980s revealed that large parts of the mitochondrial DNA (mtDNA), which code for a number of essential genes, are deleted, leading to mitochondrial deficiency. Similar age-related mtDNA reorganizations were subsequently found in other fungi and other organisms from yeast to humans [1,2,6,8,14,15,16]. More recently, studies with *P. anserina* revealed the strong impact of different mitochondrial quality control pathways in the control of aging and lifespan. For instance, control of a subset of mitochondrial proteins by mitochondrial proteases like PaLON, PaIAP or PaCLPP was demonstrated to be efficient in the control of mitochondrial homeostasis and aging [17,18,19]. Also, mitochondrial dynamics, the fission and fusion of mitochondria, which gives rise to specific mitochondrial morphotypes, was found to be involved in the control of functional mitochondria and aging [18]. More recently, a pronounced alteration in the ultrastructure of mitochondria was first identified in a short-lived mutant of *P. anserina* that overexpresses the gene encoding mitochondrial peptidyl prolyl-cis, trans-isomerase cyclophilin D (CYPD) [20]. This protein is a regulator of the mitochondrial permeability transition pore (mPTP) and involved in the control of PCD [21,22,23]. Subsequently, the same transition of mitochondria with typical tubular cristae to those in which the inner membrane forms vesicles was found to occur during normal aging of *P. anserina* cultures [24]. This process was suggested to be linked to an age-related dissociation of F_1_F_o_-ATP-synthase dimers, which is essential for the formation of the typical convex curvature at the cristae tips [25]. Recently, intervention in the F_1_F_o_-ATP-synthase dimerization process via the ablation of the F_1_F_o_-ATP-synthase assembly factor PaATPE experimentally supported this scenario. Mitochondria of the corresponding mutant displayed a vesicular ultrastructure, impairments in function and were short-lived [26].

In the present study, we set out to unravel the mechanistic basis leading to the observed acceleration of aging in the F_1_F_o_-ATP-synthase dimerization mutant of *P. anserina*. We show that lifespan reduction partially results from increased mitophagy and depends on the presence of PaCYPD. Our observations identify mitophagy as part of autophagy dependent cell death (ADCD) and a cellular response to the deleterious alteration of mitochondrial ultrastructure.

## 2. Materials and Methods

### 2.1. P. anserina Strains and Cultivation

In this study, the *P. anserina* wild-type strain “s” [27], *ΔPaAtpe* [26], *PaSod1::Gfp* [28], *PaSod3^H26L^::Gfp* [29] as well as *ΔPaAtg1* [30] and the newly generated mutants *ΔPaAtpe*/*PaSod1::Gfp*, *ΔPaAtpe*/*PaSod3^H26L^::Gfp*, *ΔPaAtpe*/*ΔPaAtg1*, *ΔPaAtpe*/*ΔPaCypD*, *ΔPaAtpe*/*ΔPaCypD*/*PaSod3^H26L^::Gfp* were used. All transgenic strains are in the genetic background of the wild-type strain s. Strains were grown on standard cornmeal agar (BMM) at 27 °C under constant light [31]. For spore germination standard cornmeal agar (BMM) supplemented with 60 mM ammonium acetate (Merck, Darmstadt, Germany; 1116.1000) was used and incubated at 27 °C in darkness for 2 days. All strains used in this study were derived from monokaryotic ascospores [31].

### 2.2. Generation of P. anserina Mutants

For double mutant generation the single mutant strains were crossed with each other. Subsequently, strains were selected from the progeny containing both mutations.

### 2.3. Southern Blot Analysis

DNA isolation was performed according to the protocol of Lecellier and Silar [32]. DNA digestion, gel electrophoresis and Southern blotting were carried out by standard protocols. According to the manufacturer’s protocol for Southern blot hybridization and detection, digoxigenin-labeled hybridization probes (DIG DNA Labeling and Detection Kit, Roche Applied Science, Mannheim, Germany, 11175033910) were used. The phleomycin resistance gene (*Ble*) specific hybridization probe corresponded to the 1293 bp BamHI-fragment of the plasmid pKO4 [17]. The 736 bp XhoI-fragment of the plasmid pSM4 [28] was used as a specific hybridization probe for the hygromycin resistance gene (*Hph*).

### 2.4. Growth Rate and Lifespan Determination

Determination of the growth rate and lifespan was performed as described in [31]. Briefly, small mycelium pieces of *P. anserina* two-day old cultures, which developed from germinated ascospores, were placed on one site of a M2 agar plates and incubated at 27 °C and constant light. When the growth front reached the end of the agar plate, a small piece of mycelium was cut out and transferred to a fresh agar plate. Growth was recorded every day until it stopped. The lifespan of *P. anserina* is defined as the time period in days (d) of linear hyphal growth while the growth rate is defined as the measured growth (cm) per time period (d).

The impact of cyclosporin A (CsA; Sigma Aldrich, St. Louis, Missouri, USA; C3805) on lifespan was performed on M2 medium supplemented with 0.025 µg/mL cyclosporin A (CsA) dissolved in ethanol (EtOH).

### 2.5. Isolation of Mitochondria

*P. anserina* strains were grown on cellophane foil-covered solid M2 agar plates for two days at 27 °C and constant light. Grown mycelia were transferred to CM-liquid medium for additional two days of growth at 27 °C and constant light. Mitochondria of *P. anserina* cultures were isolated as previously described by differential centrifugation for western blot analysis [31].

### 2.6. Isolation of Total Protein Extract

For extraction of total protein extracts, mycelia from *P. anserina* strains were cultivated for two days on cellophane foil-covered M2 agar plates under constant light and at 27 °C. Grown mycelia (up to 250 mg) were transferred into a tube filled with glass beads (Precellys Lysing Kit, Bertin Technologies, Montigny-le-Bretonneux, France; KT0393-1-004.2) and mixed with 2 volumes of protein extraction buffer containing 1 mM EDTA (Merck, Darmstadt, Germany; 1.08418.1000), 20 mM HEPES (Serva, Heidelberg, Germany; 25245), 5 mM DTT (Carl Roth, Karlsruhe, Germany; 6908.4), pH 7.5 (NaOH; Carl Roth, Karlsruhe, Germany; 6771.1). Subsequently, mycelia were homogenized two times for 25 s and 5800 rpm in a homogenizer (Precellys 24-Dual Homogenisator, Bertin Technologies, Montigny-le-Bretonneux, France), with a 10 s rest between the steps followed by a final centrifugation for 5 min at 9300× *g* at 4 °C. The supernatant was used as total protein extract.

### 2.7. Western Blot Analysis

Total protein extracts (50 µg) were separated by 2-phase SDS-PAGE (12% separating gels) according to the standard protocol [20]. After electrophoresis, protein transfer to PVDF membranes was performed with the Trans-Blot^®^ Turbo^TM^ transfer system (BIO-RAD, Hercules, CA, USA) and following the manufacturer’s specifications. Afterwards, blocking, antibody incubation and washing steps were performed according to the Odyssey “Western Blot Analysis’ handbook (LI-COR Biosciences, Bad Homburg, Germany). A number of primary antibodies were used: a PaPRX specific synthetic peptide ([Ac]-LHESSPGNKVNLADC-[NH2], New England Peptide, Gardner, MA, USA) corresponding to amino acids 43–56 (dilution: 1:2000), a monoclonal anti-GFP (mouse, 1:10,000 dilution, Sigma-Aldrich, St. Louis, MO, USA, G6795), a polyclonal anti-SOD1 (rabbit; dilution 1:5000; #SOD-100; Biomol Stressgen, Hamburg Germany), a polyclonal anti-SOD3 (rabbit; dilution 1:2000; #SOD-111; Biomol Stressgen, Hamburg Germany) and a PaSOD2 specific synthetic peptide ([Ac]-CERFLGTSEATKL[OH]; New England Peptide, Gardner, Massachusetts, USA) corresponding to the amino acids 225–236 (dilution 1:2000). Subsequently, a conjugated IR Dye CW 800 (1:15,000 dilution, goat anti-mouse 800: LIC-OR Biosciences, Bad Homburg, Germany) or IR Dye 680 RD (dilution: 1:15,000; goat anti-rabbit 680: LIC-OR Biosciences, Bad Homburg, Germany) were used as secondary antibodies. For detection, the Odyssey^®^ Fc imaging system (LIC-OR Biosciences, Bad Homburg, Germany) was used and densitometric quantification was performed with the manufacturer’s software image studio.

### 2.8. Hydrogen Peroxide Release Measurements

The hydrogen peroxide release was measured according to a protocol developed by Munkres [33]. Briefly, mycelia from different *P. anserina* strains were cultivated for four days on M2 agar plates at 27 °C under constant light. Subsequently, round pieces of the agar covered with mycelium were punched out from the plate with a 0.2 mL microcentrifuge tube and transferred to a 96-well microwell plate and overlaid with 200 µL of DAB staining solution (2.5 mM diaminobenzidine (Sigma-Aldrich St. Louis, MO, USA; D8001), 0.02 mg/mL horseradish peroxidase (Sigma-Aldrich St. Louis, MO, USA; P8250), 100 mM Tris (Carl Roth, Karlsruhe, Germany; 4855.3), pH 6.9). After incubation for 3 h at 27 °C in darkness, 100 µL of the staining solution was transferred to a fresh microplate and the absorption was measured at 471 nm in a microplate reader (Safire 2, Tecan, Maennedorf, Switzerland). For each strain, at least ten different monokaryotic ascospore isolates (biological replicates) were used and three pieces of mycelium (technical replicates) from each isolate were measured. The absorption values were normalized with the dry weight of the mycelium. Therefore, additional pieces of agar covered with mycelium (same size as those used for measurement of hydrogen peroxide release) were punched out of the agar and boiled in water for 1.5 min to remove the agar. After filtration and drying the weight was determined.

### 2.9. In-Gel SOD Activity Assay

For the in-gel SOD activity assay of mitochondrial (50 µg) or total protein (100 µg), protein extracts were loaded on two native polyacrylamide gels (10% separating gels and 5% stacking gels). Electrophoresis was performed for 1.5 h at 100 V. One gel was used for the SOD activity staining with nitro blue tetrazolium (Sigma-Aldrich, St. Louis, MO, USA; N6876), riboflavin (Sigma-Aldrich, St. Louis, MO, USA; R4500) and TEMED (Carl Roth, Karlsruhe, Germany; 2367.1) as described in [34].

### 2.10. In-Gel Peroxidase Activity Assay

The basic procedure was adapted from a previously published protocol [35]. Total protein extract (30 μg) was loaded on two 10% native gels. Electrophoresis was performed for 16 h at 70 V and 4 °C. Subsequently, one gel was used for the peroxidase activity staining and the other gel acted as a loading control by Coomassie staining. For the peroxidase activity detection, the gel was washed three times for 10 min in 1× PBS (pH 7.4). Afterwards the gel was incubated in 30 mL DAB-solution (30 mg diaminobenzidine (Sigma-Aldrich, St. Louis, MO, USA; D8001) dissolved in 30 mL 1× PBS, pH 7.4) and 3 μL 30% hydrogen peroxide (Carl Roth, Karlsruhe, Germany; 8070.2) for 20 min on a rocking platform. To stop the reaction, the gel was washed two times in H_2_O. The result was documented by scanning the gel.

### 2.11. Fluorescence Microscopy

For microscopic analysis, *P. anserina* strains were cultivated on glass slides with a central depression containing M2 medium for 1 day at 27 °C and constant light. For vacuole staining, the strains were treated with 2 μg/mL FM^TM^ 4–64 Dye (Invitrogen, Waltham, MA, USA; T13320) 5 h before microscopic analysis and was transferred to darkness. For visualization and documentation of hyphae, a fluorescence microscope (DM LB/11888011, Leica, Wetzlar, Germany) equipped with the appropriate excitation and emission filters was used.

### 2.12. Statistical Analysis

Lifespans were statistically analyzed with the IBM SPSS statistics 19 software package (IBM, Armonk, NY, USA) by generating the Kaplan-Meier survival estimates. Significances were determined with pairwise comparison with three independent statistical tests (Breslow (generalized Wilcoxon), log-rank (Mantel–Cox), and the Tarone–Ware). For a better overview, only the *p*-value calculated with the Breslow test is given in the legends. All other values can be found in the Supplementary Materials (Table S1). In addition, statistical analyses were performed with the two-tailed Student’s *t*-test. For statistical significance a minimum threshold of *p* ≤ 0.05 was set. *p* ≤ 0.05: *, *p* ≤ 0.01: **, *p* ≤ 0.001: ***.

## 3. Results

### 3.1. Defects in F_1_F_o_-ATP-Synthase Dimerization Leads to an Increased Hydrogen Peroxide Release

A previous study revealed that deletion of a gene coding for the PaATPE assembly factor impairs F_1_F_o_-ATP-synthase dimerization and mitochondrial functions, increases hydrogen peroxide release of cultures and accelerates organismic aging [26]. In the current study, we set out to shed more light on the mechanistic basis of this process. A crucial factor is hydrogen peroxide, a membrane permeable reactive oxygen species (ROS) that is involved in oxidative damage and in ROS signaling. In particular, it is known that hydrogen peroxide is able to induce autophagy, the vacuolar degradation of cellular components [36].

**Figure 1:**
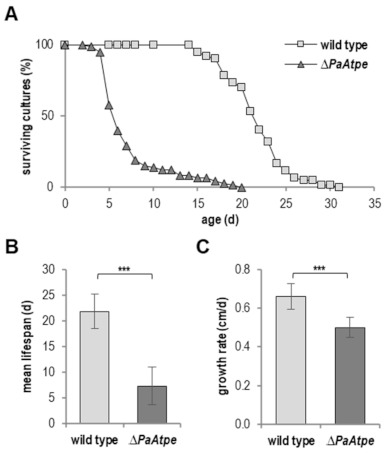
Deficiency in F_1_F_o_-ATP-synthase dimerization leads to lifespan and growth rate reduction. (**A**) Survival curve of *P. anserina* wild type (*n* = 60) and *ΔPaAtpe* (*n* = 73; *p* = 1.5 × 10^−25^) grown on standard M2 medium. (**B**) Mean lifespan and (**C**) mean growth rates of cultures from (**A**). Error bars correspond to the standard deviation. The *p*-values of the survival curves were determined by SPSS with three different tests, and those for mean lifespan and growth rate by two-tailed Student’s *t* test. *** *p* < 0.001.

For practical reasons, we performed experiments on agar plates. Therefore, we first analyzed whether the effects on lifespan obtained in race tubes in the earlier study [26] also occur on agar plates. We found the mean lifespan was reduced by 66.5% (21.8 d vs. 7.3 d) and the maximal lifespan by 35.5% (20 d vs. 31 d) (Figure 1A,B), which was even more pronounced than in race tubes. Also, as expected from the initial study, the growth rate of the *PaAtpe* deletion mutant was reduced (Figure 1C).

**Figure 2:**
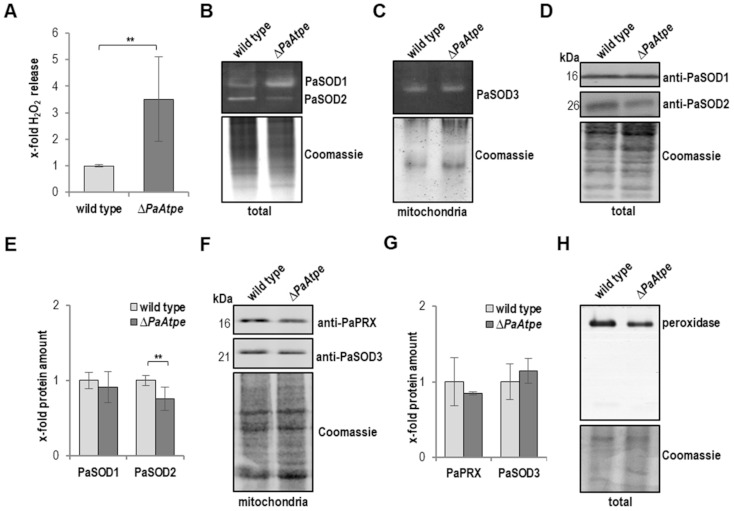
ROS metabolism is affected by the loss of F_1_F_o_-ATP-synthase dimers. (**A**) Quantitative measurement of hydrogen peroxide (H_2_O_2_) release from *P. anserina* wild type (*n* = 11) and *ΔPaAtpe* (*n* = 10). The mean release of hydrogen peroxide of the wild type was set to 1. (**B**) Representative superoxide dismutase (SOD) (PaSOD1 and PaSOD2) activity gel from total protein extracts of *P. anserina* wild type and *ΔPaAtpe* (each 4 biological replicates). (**C**) Representative superoxide dismutase (PaSOD3) activity gel from mitochondrial protein extract of *P. anserina* wild type and *ΔPaAtpe* (each 3 biological replicates). (**D**) Western blot analysis of total protein extract of *P. anserina* wild type and the *ΔPaAtpe* mutant (each 4 biological replicates). (**E**) Quantification of PaSOD1 and PaSOD2 protein abundance normalized to the Coomassie stained gel. Protein level in wild-type cultures was set to 1. (**F**) Western blot analysis of mitochondrial protein extract of *P. anserina* wild type and *ΔPaAtpe* (each 3 biological replicates). (**G**) Quantification of PaPRX and PaSOD3 protein levels normalized to the Coomassie stained gel. Protein level in wild-type cultures was set to 1. (**H**) Representative peroxidase activity gel from total protein extracts of *P. anserina* wild type and *ΔPaAtpe* (each 4 biological replicates). (**A**,**E**,**G**) Error bars correspond to the standard deviation and *p*-value was determined by two-tailed Student’s *t*-test. ** *p* < 0.01.

Next, we quantified the release of hydrogen peroxide, from wild-type and *∆PaAtpe* cultures using a quantitative photometric method. Consistent with the results from the qualitative histochemical analysis of the initial study [26], *∆PaAtpe* cultures released almost three times more hydrogen peroxide than those of the wild type (Figure 2A). This increase is an indirect measure of cellular ROS levels.

In the next series of experiments, we set out to determine the reason for the increase of hydrogen peroxide in *∆PaAtpe.* It may result from the increased formation or the decreased degradation of this ROS. To discriminate between these two possibilities, we investigated hydrogen peroxide producing and degrading enzymes. Using an in-gel staining assay, we examined the activities of the superoxide dismutase (SOD) isoforms of *P. anserina* (Figure 2B,C), which, via the disproportion of the superoxide anion, lead to the generation of hydrogen peroxide. In comparison to wild-type strains, we found an increase of the cytosolic PaSOD1 activity in *∆PaAtpe* strains (Figure 2B). In contrast, PaSOD2 activity, a secreted SOD of *P. anserina* [28] (Figure 2B), was decreased and mitochondrial PaSOD3 activity did not change (Figure 2C). In contrast, PaSOD2 activity, a SOD located in the perinuclear endoplasmatic reticulum of *P. anserina* and secreted out of the hyphae [28] (Figure 2B), was decreased, while the mitochondrial PaSOD3 activity did not change (Figure 2C). These data raised questions about the abundance of the SODs. A western blot analysis revealed no changes in the PaSOD1 abundance (Figure 2D,E) although the activity of the enzyme was increased. The data are in agreement with the earlier finding that PaSOD1 is post-translationally regulated via the incorporation of copper as the co-factor [37,38]. Thus, it is well possible that cytoplasmic copper levels, as is known to occur during normal aging of *P. anserina* [39], may be increased in the mutant. At this stage of our study, we have not investigated this issue further. Concerning the activity of PaSOD2, activity reduction goes along with a significant decrease in protein abundance (Figure 2D,E). Also, the data from the activity analysis of PaSOD3 isoform correspond to the abundance of the protein (Figure 2F,G). They were both unchanged in the mutant compared to the wild type.

Subsequently, we analyzed the degradation of hydrogen peroxide by scavenging enzymes. Compared to the wild type, western blot analysis revealed a slight decrease in the peroxiredoxin protein abundance (Figure 2F,G). Furthermore, peroxidase activity determined by in-gel staining was decreased in *∆PaAtpe* cultures (Figure 2H). Overall, these data demonstrated that both the increased formation and decreased degradation of hydrogen peroxide contribute to the enhanced hydrogen peroxide release from *∆PaAtpe* cultures.

### 3.2. Loss of F_1_F_o_-ATP-Synthase Dimers Leads to Increased Vacuolization and Mitophagy Induction

**Figure 3:**
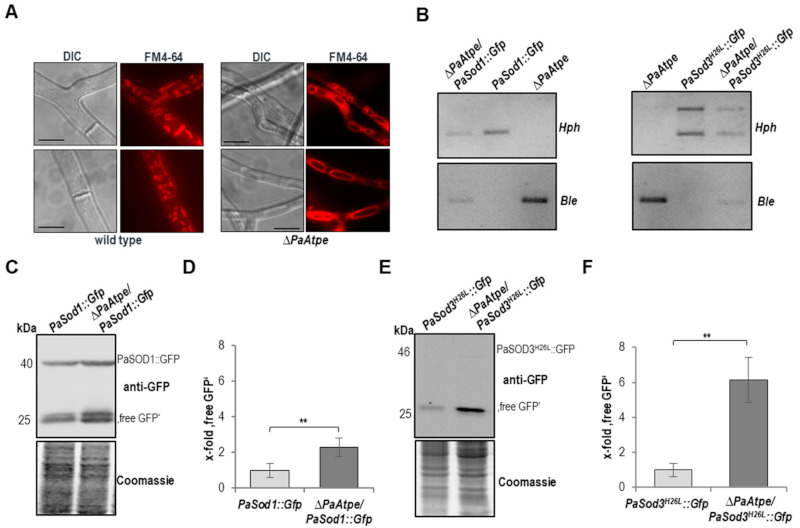
Loss of F_1_F_o_-ATP-synthase dimers induced autophagic processes (**A**) Fluorescence microscopy of 6-day-old *P. anserina* wild-type and *ΔPaAtpe* strains after cultivation for one day before microscopic analysis on M2 medium. Staining of vacuole membrane with 2 µg/mL FM4-64 for 5 h. Scale bars: 10 μm. (**B**) Southern blot analyses verify the newly generated double mutants *ΔPaAtpe/PaSod1::Gfp* and *ΔPaAtpe/PaSod3^H26L^::Gfp* by the phleomycin (*Ble*) and the hygromycin (*Hph*) resistance genes. (**C**) Monitoring non-selective autophagy by western blot analysis of total protein extract from *PaSod1::Gfp* and *∆PaAtpe/PaSod1::Gfp* cultures with a GFP-antibody (each 6 biological replicates). (**D**) Quantification of “free GFP” protein level from (C) normalized to the Coomassie stained gel. Protein level in *PaSod1::Gfp* cultures were set to 1. (**E**) Monitoring mitophagy by western blot analysis of total protein extract from *PaSod3^H26L^::Gfp* and *∆PaAtpe/PaSod3^H26L^::Gfp* cultures with a GFP-antibody (each 4 biological replicates). (**F**) Quantification of “free GFP” protein level from (E) normalized to the Coomassie stained gel. Protein level in *PaSod3^H26L^::Gfp* cultures were set to 1. Error bars correspond to the standard deviation and *p*-values were determined by two-tailed Student’s *t* test. ** *p* < 0.01.

Mitochondrial dysfunction and fragmentation as well as increased hydrogen peroxide levels have been reported to induce autophagic processes [36,40,41,42,43,44,45,46]. These possible inducers occur in the *PaAtpe* deletion mutant. Consequently, we investigated whether or not there are signs for increased autophagic processes. First, we stained hyphae of the wild type and *ΔPaAtpe* from 6-days old cultures with FM4-64, a dye that localizes to vacuolar membranes (Figure 3A). We found that hyphae of the mutant were smaller in size than those of the wild type in which small vesicles were observed. Also, no vacuoles were visible in differential interference contrast (DIC) images. In contrast, mutant vacuoles were found in DIC. FM4-64 staining visualized this larger number of vacuoles, suggesting autophagy was induced in the *PaAtpe* deletion mutant.

In order to demonstrate autophagy in *ΔPaAtpe* and to discriminate between non-selective and selective autophagy, we performed a biochemical analysis using GFP-cleavage-assay. In this assay, vacuolar degradation of marker proteins fused to GFP was monitored by detection of the degradation-resistant GFP-part (“free GFP”), indicating ongoing autophagy. To determine non-selective autophagy, in which the autophagosomal membrane non-selectively encloses a portion of the cytoplasm, we used PaSOD1::GFP fusion protein as a cytoplasmic marker protein [30]. To measure mitophagy, the selective vacuolar degradation of mitochondria, we used the mitochondrial PaSOD3^H26L^::GFP fusion protein [29] (Figure 3B). Western blot analysis of total protein extracts revealed a 2-fold increase in the amount of “free GFP” derived from the non-selective autophagy reporter PaSOD1::GFP (Figure 3C,D). The analysis of “free GFP” resulting from degradation of the mitophagy reporter PaSOD3^H26L^::GFP displayed a 6-fold increase in the deletion strain compared to the wild type (Figure 3E,F). Noticeably, the lack of a detectable amount of fusion protein PaSOD3^H26L^::GFP was due to the very low abundance of mitochondrial proteins in total protein extracts. However, as demonstrated in earlier studies, the fusion protein was clearly visible in mitochondrial extracts [29]. The mitophagy reporter may also be degraded together with cytosolic compounds unspecifically by non-selective autophagy to some extent because some mitochondria are part of the cytoplasmic portion engulfed by the autophagosomal membrane. However, since the increase in degradation of PaSOD3^H26L^::GFP was much higher than the increase in PaSOD1::GFP degradation (6-fold vs. 2-fold) a preferential degradation of PaSOD3^H26L^::GFP by mitophagy can be concluded. Overall, these data indicate an induction of both non-selective autophagy and mitophagy, with a much stronger induction of the latter in *ΔPaAtpe* strains.

### 3.3. Mitophagy Induction Contributes to Lifespan Decrease of ΔPaAtpe

**Figure 4:**
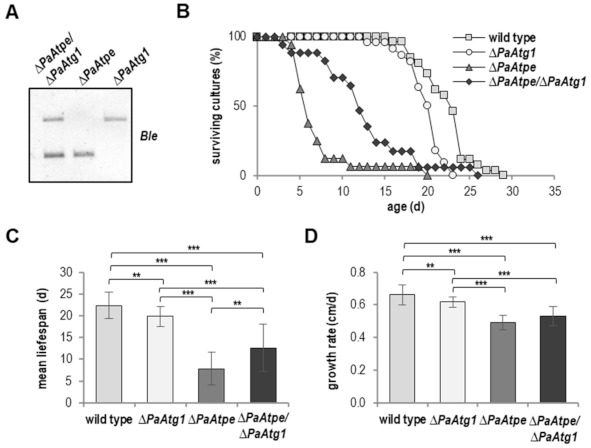
Autophagic processes contributes to lifespan decrease of *ΔPaAtpe*. (**A**) Southern blot analysis verifies the newly generated double deletion mutant *ΔPaAtpe/ΔPaAtg1* by the phleomycin (*Ble*) resistance genes (**B**) Survival curve of *P. anserina* wild type (*n* = 26), *ΔPaAtg1* (*n* = 22; *p* = 2.6 × 10^−3^), *ΔPaAtpe* (*n* = 16; *p* = 7.5 × 10^−11^) and *ΔPaAtpe/ΔPaAtg1* (*n* = 17; *p* = 2.6 × 10^−8^) grown on standard M2 medium. (**C**) Mean lifespan and (**D**) mean growth rates of cultures from (B). Error bars correspond to standard deviation. The *p*-values of the survival curves were determined by SPSS with three different tests and for mean lifespan and growth rate by two-tailed Student’s *t* test. ** *p* < 0.01; *** *p* < 0.001.

Autophagy acts as a double-edged sword. At low and moderate levels, autophagy has a pro-survival effect while excess autophagy has a pro-death function [47]. This also applies for autophagy in *P. anserina* where it was shown that autophagic processes can compensate mitochondrial impairments while excessive autophagy is detrimental and leads to lifespan reduction [29,48,49]. To discriminate between these two functions of autophagy in *ΔPaAtpe*, we generated and analyzed the mutant in a genetic background in which autophagy is blocked (Figure 4A). The generation of the corresponding *ΔPaAtpe/ΔPaAtg1* double mutant was difficult because most isolated spores did not germinate due to impaired fertility, a spore germination defect of *ΔPaAtg1* [30], as well as the very short lifespan of *ΔPaAtpe.* In comparison to the *ΔPaAtpe* single knockout mutant, the lifespan of the *ΔPaAtpe/ΔPaAtg1* mutant was strongly increased, although it did not reach the lifespan of the wild type or that of the *ΔPaAtg1* mutant (Figure 4B). In the double mutant, mean lifespan was increased by 61.5% (12.6 d vs. 7.8 d) and maximal lifespan by 45% (29 d vs. 20 d) (Figure 4B,C). Compared to *ΔPaAtpe,* the growth rate of the double deletion mutant was unchanged (Figure 4D). Taken together, these data revealed the pro-death function of mitophagy in the F_1_F_o_-ATP-synthase dimerization mutant. Hence, lifespan reduction of the *PaAtpe* deletion mutant partly depends on the induction of mitophagy.

### 3.4. Lifespan Reduction of ΔPaAtpe Is Linked to the Induction of mPTP-Mediated Mitophagy

**Figure 5:**
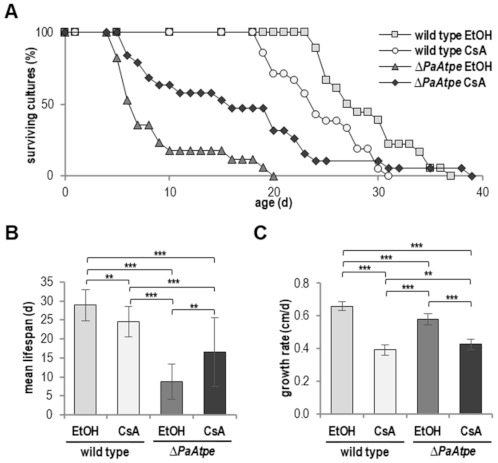
Inhibition of mPTP partially restores lifespan of *ΔPaAtpe*. (**A**) Survival curve of *P. anserina* wild type and *ΔPaAtpe* grown on standard M2 medium with ethanol (EtOH) as control or cyclosporine A (CsA) (each 18 biological replicates). (**B**) Mean lifespan and (**C**) mean growth rates of cultures from (**A**). Error bars correspond to the standard deviation. The *p*-values of the survival curves were determined by SPSS with three different tests and for mean lifespan and growth rate by two-tailed Student’s *t* test. ** *p* < 0.01, *** *p* < 0.001.

In *P. anserina* and in other organisms, mitophagy induction depends on mPTP opening [49,50,51,52], which is mediated by hydrogen peroxide [21,53,54,55]. In order to investigate the role of the mPTP in *ΔPaAtpe* we inhibited CYPD, a regulator of the mPTP, by its specific inhibitor cyclosporin A (CsA) [56,57]. Since CsA is dissolved in ethanol (EtOH), we needed to measure lifespans of all strains on medium containing EtOH. We found that the inhibition of mPTP reduced the maximal lifespan of the wild type by 16.2% (31 d vs. 37 d) and mean lifespan by 15.2% (24.5 d vs. 28.9 d) (Figure 5A,B). This is consistent with what was found in a previous study [49].

In contrast, inhibition of mPTP opening in *ΔPaAtpe* resulted in an increased lifespan (Figure 5A,B). This is similar to what we found in the *ΔPaAtpe* in which autophagy was inhibited. CsA treatment of *ΔPaAtpe* led to an increase of mean lifespan by 87.5% (16.5 d vs. 8.8 d) and a maximal lifespan increase of 95% (39 d vs. 20 d) (Figure 5A,B). In both, the wild type and the *PaAtpe* deletion mutant, the growth rate was reduced by CsA treatment (Figure 5C). The basic reduction in the growth rate of all strains on CsA-containing medium is consistent with the poisoning effect of CYPD/CsA interaction products that was previously demonstrated in the filamentous ascomycete *Neurospora crassa* [58,59].

**Figure 6:**
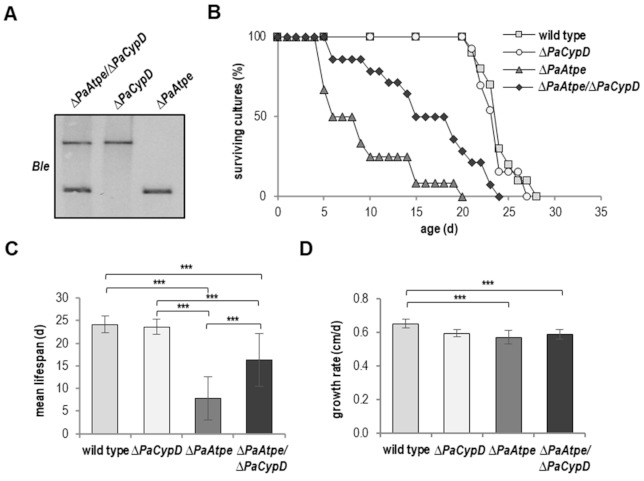
Deletion of *PaCypD* partially restores lifespan of the dimerization mutant. (**A**) Southern blot analysis verifies the newly generated double mutant *ΔPaAtpe/ΔPaCypD* by the phleomycin (*Ble*) resistance genes (**B**) Survival curve of *P. anserina* wild type (*n* = 10), *ΔPaCypD* (*n* = 13); *ΔPaAtpe* (*n* = 12; *p* = 0.00002) and *ΔPaAtpe/ΔPaCypD* (*n* = 14; *p* = 0.0004) grown on standard M2 medium. (**C**) Mean lifespan and (**D**) mean growth rates of cultures from (B). Error bars correspond to the standard deviation. The *p*-values of the survival curves were determined by SPSS with three different tests that for mean lifespan and growth rate by two-tailed Student’s *t* test. *** *p* < 0.001.

In order to avoid such side effects of the chemical inhibition by CsA, we next analyzed a newly generated *ΔPaAtpe/ΔPaCypD* double deletion mutant. In this mutant, both the *PaAtpe* and the *PaCypD* genes were replaced by a phleomycin resistance cassette (*Ble*) (Figure 6A). Strikingly, compared to the lifespan of *ΔPaAtpe* and like the chemical inhibition of PaCYPD, impairment of mPTP resulting from the ablation of PaCYPD revealed an increase in lifespan of the *ΔPaAtpe/ΔPaCypD* double deletion mutant (Figure 6B). In contrast, the lifespan of the single *PaCypD* deletion mutant was unchanged when compared to the wild type (Figure 6B). The simultaneous ablation of both genes caused a 20% enhanced maximal lifespan (24 d vs. 20 d) and a 107.7% increased mean lifespan (16.2 d vs. 7.8 d) (Figure 6B,C), indicating that lifespan reduction after ablation of PaATPE was PaCYPD-dependent, and thus, mPTP-mediated.

**Figure 7:**
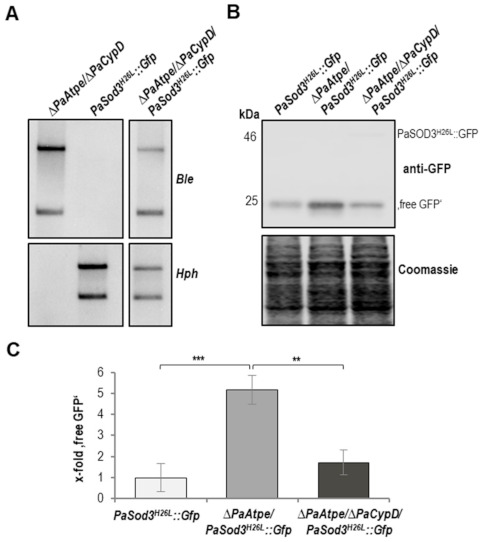
Mitophagy induction in *ΔPaAtpe* is dependent of PaCYPD. (**A**) Southern blot analysis verifies the newly generated double mutant *ΔPaAtpe/ΔPaCypD/PaSod3^H26L^::Gfp* by the phleomycin (*Ble*) and hygromycin (*Hph*) resistance genes. (**B**) Monitoring mitophagy by western blot analysis of total protein extract from *PaSod3^H26L^::Gfp*, *∆PaAtpe/PaSod3^H26L^::Gfp* and *∆PaAtpe/∆PaCypD/PaSod3^H26L^::Gfp* cultures with a GFP-antibody (each 4 biological replicates). (**C**) Quantification of “free GFP” protein levels from (B) normalized to the Coomassie stained gel. Protein level in *PaSod3^H26L^::Gfp* cultures were set to 1. Error bars correspond to the standard deviation and *p*-values were determined by two-tailed Student’s *t* test. ** *p* < 0.01, *** *p* < 0.001.

To validate this conclusion, we constructed a strain to monitor mitophagy in the *ΔPaAtpe/ΔPaCypD* double mutant. The corresponding triple mutant expresses the mitophagy reporter gene *PaSod^H26L^::Gfp* (Figure 7A). Analysis of this strain revealed that mitophagy induction in *ΔPaAtpe* strains is PaCYPD-dependent (Figure 7B,C). The amount of “free GFP” in the *ΔPaAtpe*/*ΔPaCypD*/*PaSod3^H26L^::Gfp* strains is almost the same as in the *PaSod3^H26L^::Gfp* strain with a functional *PaAtpe* gene and significantly lower than in *ΔPaAtpe* strains. These data demonstrate no mitophagy induction in the *ΔPaAtpe* in the absence of PaCYPD and support the conclusion that mitophagy induction in *ΔPaAtpe* depends on mPTP opening.

Overall, our data show a clear link between the premature onset of aging and autophagy, and specifically, mitophagy induction and demonstrate that premature death of the *ΔPaAtpe* mutant depends on mPTP-dependent autophagy induction, and as such, a form of autophagy-dependent cell death (ADCD) [60]. Since impairment of autophagy in the *ΔPaAtpe* mutant only leads to a partial reversion of the wild-type lifespan, we conclude that in addition to ADCD at least one other, not yet determined pathway, contributes to the accelerated aging observed in the *ΔPaAtpe* mutant. For instance, type-I PCD could be activated by the massive impairment of mitochondria in the *ΔPaAtpe* mutant.

## 4. Discussion

The final step in the lifetime of *P. anserina* is the induction and execution of PCD. Known components of the underlying machinery include the two metacaspases PaMCA1 and PaMCA2, apoptosis inducing factors (AIFs), poly (ADP-ribose) polymerase (PARP), cyclophilin D (CYPD) and apoptotic or type I PCD has been identified as the underlying mechanism [20,61,62,63,64]. In addition, cell death may occur via the excessive induction of autophagy leading to type II PCD or autophagy-dependent cell death (ADCD). What kind of PCD is active under specific situations or in individual mutants is complicated by the fact that individual components, like CYPD, of the molecular machinery may be active in both types of PCD. Moreover, in addition to the pro-death function of autophagy, autophagy also plays a role in the control of cellular homeostasis, and as such, it has a pro-survival function and is a longevity assurance mechanism [30,65]. In *P. anserina*, we found that this latter role is active in situations when cells are stressed as a result of nutrient starvation, increased ROS stress, or due to impairments of other quality control pathways like deficiencies in protein quality control by specific proteases [29,48]. Even more, the induction of autophagy by mild stress can lead to hormetic responses, which are beneficial in nature by counteracting degenerative processes, leading to increases in lifespan. Such processes have been demonstrated in different systems including *P. anserina*, *Saccharomyces cerevisiae*, *Drosophila melanogaster*, *Caenorhabditis elegans* and mice [29,66]. The possibility of triggering the two opposing functions of autophagy provides very promising strategies to intervene in diseases like cancer or age-related degeneration, and in this way may contribute to extending the healthy period of life, the healthspan, of biological systems including the human species. To achieve this goal, it is essential to understand those processes in detail and which are effective.

In the current study, we specifically investigated an age-related process: the extensive remodeling of the inner mitochondrial membrane. This process includes the dissociation of F_1_F_o_-ATP-synthase dimers at the tips of mitochondrial cristae and the transition of mitochondria with tubular cristae to those in which the inner membrane forms vesicles. Such age-related changes of the inner mitochondrial membrane are evolutionary conserved and were described in *P. anserina, D. melanogaster*, rodents, as well as different human cell lines [20,24,25,67,68,69]. Previously we demonstrated that the dimerization catalyzed by specific F_1_F_o_-ATP-synthase dimerization factors (ATPE and ATPG) is critical for fully functional mitochondria. Ablation of PaATPE leads to the formation of vesicular mitochondrial ultrastructure, mitochondrial impairment, increased hydrogen peroxide release and a decreased lifespan [26].

Here we show that the increases in cellular ROS resulted from both an increase in ROS generation and a reduction of ROS scavenging. As a result, an enhanced release of hydrogen peroxide from *ΔPaAtpe* cultures occurs. In general, ROS have dual functions. Increased levels lead to oxidative stress and damage of biomolecules resulting in deleterious effects. In contrast, low ROS levels are required for signal transduction and are essential for cellular homeostasis [61,70]. In the *ΔPaAtpe* mutant, increased hydrogen peroxide appears to induce both general autophagy and more strongly, mitophagy and leads to accelerated aging. Such a scenario is consistent with the knowledge that oxidative stress caused by increased hydrogen peroxide can induce autophagic processes [36,46].

In part, this effect depends on the functional autophagy machinery and on the mPTP regulator PaCYPD, and thus, links mitophagy to the mPTP of *P. anserina*. Also, the CYPD-mediated opening of the mPTP was previously shown to be induced by ROS [21,53,54,55]. Since the ablation of components of this machinery or their inhibition only partially restores the wild-type lifespan of the *ΔPaAtpe* deletion strain, it appears that in addition to ADCD, at least another yet unknown pathway is responsible for premature aging of this mutant. Here, a pathway involved in the control of type-I PCD may be active.

Significantly, the identified link between CYPD-mediated mitophagy induction and cell death in the *ΔPaAtpe* mutant was previously also uncovered in a *P. anserina* mutant in which *PaCypD* is strongly overexpressed [49]. In this study, permanent mPTP opening was found to be responsible for an excessive induction of mitophagy, and thus, for premature cell death. Like the *ΔPaAtp*e mutant, the *PaCypD* overexpressor is characterized by an impaired mitochondrial ultrastructure and function [20]. Interestingly, this mechanism is not unique to *P. anserina*. Recently, in human glioma cells, which are deficient in type-I PCD, the polyphenol gossypol (AT 101) triggered an mPTP-mediated mitophagy-dependent type of cell death [46,71].

Overall, the results of the current study demonstrate a novel link between mitochondrial ultrastructure remodeling and mPTP-mediated induction of ADCD, and integrate this type of PCD into the complex molecular network involved in the control of cellular homeostasis and lifespan. The induction of autophagy-dependent responses can be triggered by the application of exogenous molecules like the natural polyphenols, curcumin and gossypol. The affected molecular mechanisms appear to be evolutionary conserved. Targeting them with specific compounds is a promising strategy to intervene in diseases like cancer or age-related degeneration. In this way they may contribute to extending the healthspan of biological systems including the human species.

## Data Availability

The data presented in this study are available on reasonable request from the corresponding author.

## Supplementary Materials

The following are available online at https://www.mdpi.com/article/10.3390/cells10040757/s1, Table S1: *p*-values of survival curve analysis with SPSS. Sample sizes are given in the brackets.
