# Supplementary material for: Impaired F1Fo-ATP-Synthase Dimerization Leads to the Induction of Cyclophilin D-Mediated Autophagy-Dependent Cell Death and Accelerated Aging
Source: Cells. 2021 Mar 30;10(4):757. doi: 10.3390/cells10040757 (PMC8066942; doi:10.3390/cells10040757)
Supplement: Supplementary file 1 [file cells-10-00757-s001.pdf]

**Table** S**1.** *p*-values of survival curve analysis with SPSS. Sample sizes are given in the brackets.

| | | **Long Rank** | **Breslow** | **Tarane-Ware** |
|---|---|---|---|---|
| Figure 1 | *ΔPaAtpe* vs. WT (60 vs. 73) | $3.4 \times 10^{-31}$ | $1.5 \times 10^{-25}$ | $1.9 \times 10^{-28}$ |
| Figure 4 | *ΔPaAtpe* vs. WT (16 vs. 26) | $8.1 \times 10^{-12}$ | $7.5 \times 10^{-11}$ | $2.2 \times 10^{-11}$ |
| | *ΔPaAtg1* vs. WT (22 vs. 26) | $2.3 \times 10^{-4}$ | $2.6 \times 10^{-3}$ | $8.4 \times 10^{-4}$ |
| | *ΔPaAtpe/ΔPaAtg1* vs. *WT* (17 vs. 26) | $3.9 \times 10^{-7}$ | $2.6 \times 10^{-8}$ | $5.6 \times 10^{-8}$ |
| | *ΔPaAtpe* vs. *ΔPaAtg1* (16 vs. 22) | $2.3 \times 10^{-9}$ | $5.7 \times 10^{-9}$ | $2.9 \times 10^{-9}$ |
| | *ΔPaAtpe/ΔPaAtg1* vs. *ΔPaAtpe* (17 vs. 16) | $3.0 \times 10^{-3}$ | $1.0 \times 10^{-3}$ | $1.0 \times 10^{-3}$ |
| | *ΔPaAtpe/ΔPaAtg1* vs. *ΔPaAtg1* (17 vs. 22) | $2.4 \times 10^{-4}$ | $2.2 \times 10^{-6}$ | $8.5 \times 10^{-6}$ |
| Figure 5 | WT CsA vs. WT EtOH (21 vs. 18) | $3.7*10^{-3}$ | $3.8 \times 10^{-3}$ | $3.8 \times 10^{-3}$ |
| | *ΔPaAtpe* EtOH vs. WT EtOH (17 vs. 18) | $1.6 \times 10^{-10}$ | $7.2 \times 10^{-9}$ | $1.1 \times 10^{-9}$ |
| | *ΔPaAtpe* CsA vs. WT EtOH (19 vs. 18) | $8.3 \times 10^{-5}$ | $4.3 \times 10^{-7}$ | $1.3 \times 10^{-6}$ |
| | *ΔPaAtpe* CsA vs. *ΔPaAtpe* EtOH (19 vs. 17) | $5.1 \times 10^{-3}$ | $1.4 \times 10^{-2}$ | $8.2 \times 10^{-3}$ |
| Figure 6 | *ΔPaAtpe* vs. WT (12 vs. 10) | $1.8 \times 10^{-6}$ | $1.7 \times 10^{-5}$ | $5.5 \times 10^{-6}$ |
| | *ΔPaCypD* vs. WT (13 vs. 10) | $5 \times 10^{-1}$ | $4.4 \times 10^{-1}$ | $4.6 \times 10^{-1}$ |
| | *ΔPaAtpe/ΔPaCypD* vs. *WT* (14 vs. 10) | $3.0 \times 10^{-4}$ | $3.8 \times 10^{-4}$ | $3.0 \times 10^{-4}$ |
| | *ΔPaAtpe* vs. *ΔPaCypD* (12 vs. 13) | $1.0 \times 10^{-7}$ | $1.2 \times 10^{-6}$ | $3.4 \times 10^{-7}$ |
| | *ΔPaAtpe/ΔPaCypD* vs. *ΔPaAtpe* (14 vs. 12) | $4.4 \times 10^{-3}$ | $3.4 \times 10^{-3}$ | $3.5 \times 10^{-3}$ |
| | *ΔPaAtpe/ΔPaCypD* vs. *ΔPaCypD* (14 vs. 13) | $3.5 \times 10^{-4}$ | $2.2 \times 10^{-4}$ | $2.4 \times 10^{-4}$ |
